# Reliability of HR-pQCT Derived Cortical Bone Structural Parameters When Using Uncorrected Instead of Corrected Automatically Generated Endocortical Contours in a Cross-Sectional Study: The Maastricht Study

**DOI:** 10.1007/s00223-018-0416-2

**Published:** 2018-03-29

**Authors:** Ellis A. C. de Waard, Cindy Sarodnik, Alexander Pennings, Joost J. A. de Jong, Hans H. C. M. Savelberg, Tineke A. van Geel, Carla J. van der Kallen, Coen D. A. Stehouwer, Miranda T. Schram, Nicolaas Schaper, Pieter C. Dagnelie, Piet P. M. M. Geusens, Annemarie Koster, Bert van Rietbergen, Joop P. W. van den Bergh

**Affiliations:** 1 Department of Internal Medicine, Subdivision of Rheumatology, Maastricht University, P.O. Box 616, 6200 MD Maastricht, The Netherlands; 2 NUTRIM School for Nutrition and Translational Research in Metabolism, Maastricht University, Maastricht, The Netherlands; 3 Department of Human Movement Science, Maastricht University, Maastricht, The Netherlands; 4 CAPHRI Care and Public Health Research Institute, Maastricht University, Maastricht, The Netherlands; 5 Department of Family Medicine, Maastricht University, Maastricht, The Netherlands; 6 Department of Internal Medicine, Maastricht University Medical Center, Maastricht, The Netherlands; 7 CARIM School for Cardiovascular Diseases, Maastricht University, Maastricht, The Netherlands; 8 Heart and Vascular Center, Maastricht University Medical Centre, Maastricht, The Netherlands; 9 Department of Internal Medicine, Subdivision of Rheumatology, Maastricht University Medical Centre, Maastricht, The Netherlands; 10 Biomedical Research Institute, University of Hasselt, Hasselt, Belgium; 11 Department of Social Medicine, Maastricht University, Maastricht, The Netherlands; 12 Faculty of Biomedical Engineering, Eindhoven University of Technology, Eindhoven, The Netherlands; 13 Department of Internal Medicine, Subdivision of Endocrinology, VieCuri Medical Center, Venlo, The Netherlands

**Keywords:** High resolution peripheral quantitative computed tomography, Bone microarchitecture, Cortical bone analysis, Endocortical contour

## Abstract

Most HR-pQCT studies examining cortical bone use an automatically generated endocortical contour (AUTO), which is manually corrected if it visually deviates from the apparent endocortical margin (semi-automatic method, S-AUTO). This technique may be prone to operator-related variability and is time consuming. We examined whether the AUTO instead of the S-AUTO method can be used for cortical bone analysis. Fifty scans of the distal radius and tibia from participants of The Maastricht Study were evaluated with AUTO, and subsequently with S-AUTO by three independent operators. AUTO cortical bone parameters were compared to the average parameters obtained by the three operators (S-AUTOmean). All differences in mean cortical bone parameters between AUTO and S-AUTOmean were < 5%, except for lower AUTO cortical porosity of the radius (− 16%) and tibia (− 6%), and cortical pore volume (Ct.Po.V) of the radius (− 7%). The ICC of S-AUTOmean and AUTO was > 0.90 for all parameters, except for cortical pore diameter of the radius (0.79) and tibia (0.74) and Ct.Po.V of the tibia (0.89), without systematic errors on the Bland–Altman plots. The precision errors (RMS-CV%) of the radius parameters between S-AUTOmean and AUTO were comparable to those between the individual operators, whereas the tibia RMS-CV% between S-AUTOmean and AUTO were higher than those of the individual operators. Comparison of the three operators revealed clear inter-operator variability. This study suggests that the AUTO method can be used for cortical bone analysis in a cross-sectional study, but that the absolute values—particularly of the porosity-related parameters—will be lower.

**Electronic supplementary material:**

The online version of this article (10.1007/s00223-018-0416-2) contains supplementary material, which is available to authorized users.

## Introduction

The geometry and density of the cortex of long bones are important determinants of bone strength. Since most of the bone mass lost with age is cortical, fractures at advanced age occur often at sites that mainly consist of cortical bone [1–3]. Additionally, it has been shown that cortical porosity is a good predictor of bone strength [4–6]. High resolution peripheral quantitative computed tomography (HR-pQCT) is a non-invasive three-dimensional imaging modality that has the ability to measure volumetric bone mineral density (vBMD) and microarchitecture of the cortical and trabecular region [7]. Furthermore, HR-pQCT images can be used in micro-finite element analyses (µFEA) to calculate bone strength indices [8].

Identification of the cortical region on HR-pQCT images is challenging; the transition from endocortical to trabecular bone is gradual, so no single voxel identifies the end of the cortex and the beginning of the medullary canal with its trabecular content [9]. Currently, studies examining HR-pQCT-derived cortical bone parameters mainly use a semi-automatic method (S-AUTO) provided by the manufacturer to distinguish the cortical region from the trabecular region. With this method, an endocortical contour is generated automatically first [10, 11], and the operator then manually modifies the generated contour when it visually deviates from the apparent endocortical margin. However, this method may be prone to operator-related variability and is time consuming (approximately 1 h per scan) [12]. This is particularly problematic in large cohort studies, where image analysis will be done by several operators and data from many participants need to be analyzed.

In an in vivo scan–rescan study by Kawalilak et al. it was shown that use of the uncorrected endocortical contour instead of S-AUTO contour resulted in the same repeatability of cortical bone parameters [12]. However, it is currently unknown whether the uncorrected contour can reliably be used for the assessment of cortical bone parameters in cross-sectional studies. Additionally, the magnitude of differences in cortical parameters due to inter-operator variability in modification of the contour is unknown.

In this study, we examined whether the uncorrected automatically generated contour (AUTO) instead of the S-AUTO contour can be used for cortical bone analysis in an in vivo cross-sectional study. Therefore, cortical bone parameters were first obtained with the AUTO method, and then with endocortical contours that were corrected by three independent operators. The cortical bone parameters obtained with the AUTO method were compared to the average of the cortical bone parameters obtained with the S-AUTO method of the three independent operators (S-AUTOmean). Additionally, the cortical bone parameters obtained by the three independent operators were compared to each other. We hypothesized that the AUTO method can reliably be used for cortical bone analysis.

## Materials and Methods

### Study Population and Design

Data from The Maastricht Study, an observational prospective population-based cohort study, were used. The rationale and methodology of this study have been described previously [13]. In brief, the study focuses on the etiology, pathophysiology, complications, and comorbidities of type 2 diabetes (T2DM) and is characterized by an extensive phenotyping approach. Eligible for participation were all home-dwelling individuals aged between 40 and 75 years and living in the southern part of the Netherlands. Participants were recruited through mass media campaigns and from the municipal registries and the regional Diabetes Patient Registry via mailings. Recruitment was stratified according to known type 2 diabetes status, with an oversampling of individuals with T2DM for reasons of efficiency.

The present study includes cross-sectional data from a subset of 63 consecutive participants with normal glucose metabolism, who completed the baseline survey between September 2010 and June 2013 and returned to the research center between September 2015 and January 2016 for the HR-pQCT scan of the distal radius and tibia. Participants with a radius and/or tibia scan with severe or extreme motion artifacts (i.e., quality grade 4 or 5 [14], *n* = 11), participants with a scan with an inadequate position of the reference line (reference line not on plateau of the distal radius or distal tibia *n* = 1), and participants with extreme outliers on almost all cortical parameters (> 2 SD from mean, *n* = 1) were excluded, resulting in a final study population of 50 participants. The study has been approved by the institutional medical ethical committee (NL31329.068.10) and the Minister of Health, Welfare and Sports of the Netherlands (Permit 131088-105234-PG). All participants gave written informed consent.

### HR-pQCT Imaging

The non-dominant radius and ipsilateral tibia were scanned on a HR-pQCT scanner (XtremeCT; Scanco Medical AG, Brüttisellen, Switzerland) using the standard in vivo protocol as described in the literature [7, 15]. In case of a history of a fracture of the distal radius or tibia at that site, the contralateral site was scanned. The forearm and leg were immobilized in a carbon fiber cast. An anteroposterior scout projection of the scan site was acquired for positioning of the tomographic acquisition. A reference line was placed on the plateau of the distal radius or distal tibia. The scan started 9.5 and 22.5 mm, for the radius and tibia respectively, from the reference line in the proximal direction and spanned 9.02 mm in length. Images were reconstructed using an isotropic voxel size of 82 µm, thus resulting in 110 consecutive slices. Total scan time was 2.8 min, with each acquisition resulting in an effective dose of approximately 3 µSv. All scans were graded once (operator 1) with regard to subject motion, and scans with quality grade 4 (severe motion artifacts) or 5 (extreme motion artifacts) were repeated once [14]. Only scans with quality grade 1 to 3 (no, minor or moderate motion artifacts) were used for subsequent image analysis [16].

### HR-pQCT Image Analysis

All scans were evaluated using the standard patient evaluation protocol that was provided by the manufacturer and has been described previously in detail [17–19]. First, the periosteal contour was automatically derived and manually modified by operator 1 when contours visually deviated from the periosteal boundary [20]. The endocortical contour was automatically created using a series of automatic morphological operations to separate the trabecular and cortical volumes of interest [10], resulting in the uncorrected contour (AUTO method). Then, according to Burghardt et al. [10], when the contour visually deviated from the apparent endocortical margin, it was manually corrected (S-AUTO method). Correction of the AUTO contour was performed three times by three independent operators (operator 1 (OP1), operator 2 (OP2), and operator 3 (OP3)). All images were then analyzed using the advanced cortical evaluation protocol provided by the manufacturer [10, 11]. All three operators underwent the same training for modification of the endocortical contour.

Evaluation of the cortical region resulted in the following parameters: cortical total volume (Ct.TV, mm^3^), cortical bone volume (Ct.BV, mm^3^), cortical thickness (Ct.Th, mm), cortical vBMD (Ct.BMD, mgHA/cm^3^), cortical pore volume (Ct.Po.V, mm^3^), cortical porosity (Ct.Po, %), cortical pore diameter (Ct.Po.Dm, mm), and cortical area (Ct.Ar, mm^2^).

### Statistics

All statistical analyses were performed using the Statistical Package for Social Sciences (version 22.0; IBM, Chicago, Illinois, USA). Mean and standard deviations for all cortical bone parameters were calculated using the S-AUTO contours of OP1, OP2, OP3, and (three times) the AUTO contour. All single evaluations with the AUTO method resulted in the same result; the average of the three AUTO evaluations (AUTOmean) is thus equal to AUTO. The average of the results obtained using the S-AUTO contours of OP1, OP2, and OP3, is referred to as S-AUTOmean. The mean difference in cortical bone parameters was calculated between S-AUTOmean and AUTO, and between all individual operators. A paired samples *t* test was used to test for significant differences in mean cortical bone parameters between these pairs. Non-normally distributed variables were log transformed using the natural logarithm. The Pearson correlation coefficient© and the intraclass correlation coefficient (ICC) were calculated to measure linear dependence and the level of agreement of the cortical bone parameters between S-AUTOmean and AUTO and between all individual operators. The precision error for all cortical bone parameters was calculated as the root mean square coefficient of variation (RMS-CV%) of the three operators (OP1, OP2, and OP3), the pairs of operators (OP1 and OP2, OP1 and OP3, and OP2 and OP3), and the average of the semi-automatic method and the automatic method (S-AUTOmean and AUTO) [21]. Bland–Altman plots were provided to visualize agreement between S-AUTOmean and AUTO, and between the independent operators. The limits of agreement were calculated as the mean value ± 1.96 * SD. A *p* value < 0.05 was considered statistically significant.

## Results

Data from 50 participants with normal glucose metabolism and a HR-pQCT scan of the distal radius and tibia with quality grade 1–3 were used for analysis. The mean age of the participants was 57.3 ± 8.7 year and 60% were women. Five (10.0%) scans of the distal radius and 25 (50.0%) scans of the distal tibia were graded as quality 1, 29 (58.0%) scans of the distal radius and 18 (36.0%) scans of the distal tibia were graded as quality 2, and 16 (32.0%) scans of the distal radius and 7 (14.0%) scans of the distal tibia were graded as quality 3.

### Mean Cortical Bone Parameters

The mean cortical bone parameters obtained by the individual operators and by AUTO and the differences in mean cortical parameters between S-AUTOmean and AUTO are shown in Table 1 (radius) and Table 3 (tibia). The differences in mean cortical parameters of the pairs of operators (OP1–OP2, OP1–OP3, and OP2–OP3) are shown in Table 2 (radius) and Table 4 (tibia).

**Table 1:** Mean values, differences in mean values, correlations, and ICCs of the cortical bone parameters of the distal radius

	OP1	OP2	OP3	S-AUTOmean	AUTO	Diff between S-AUTOmean and AUTO	Diff between S-AUTOmean and AUTO (%)	Pearson’s *r* S-AUTOmean–AUTO	ICC OP1–OP2–OP3 (95% CI)	ICC S-AUTOmean–AUTO (95% CI)
Ct.TV	549.4 (138.6)	550.2 (140.8)	565.8 (142.7)	555.1 (140.5)	542.3 (140.3)	12.8* (10.8)	2.3	1.00*	0.99* (0.97–1.00)	0.99* (0.90–1.00)
Ct.BV	505.2 (136.0)	505.6 (136.0)	512.0 (137.6)	507.6 (137.0)	501.5 (137.1)	6.04* (5.26)	1.2	1.00*	1.00* (0.99–1.00)	1.00* (0.98–1.00)
Ct.Th	0.84 (0.18)	0.84 (0.18)	0.84 (0.18)	0.84 (0.18)	0.84 (0.18)	0.00 (0.01)	0.1	1.00*	1.00* (1.00–1.00)	1.00* (1.00–1.00)
Ct.vBMD	945.2 (60.7)	947.2 (57.0)	939.0 (59.1)	943.8 (58.7)	953.2 (53.6)	− 9.35* (11.3)	− 1.0	0.98*	0.98* (0.96–0.99)	0.97* (0.84–0.99)
Ct.Po.V	17.5 (10.8)	17.1 (10.2)	20.0 (11.4)	18.2 (10.7)	14.9 (9.1)					
Log Ct.Po.V	2.68 (0.62)	2.67 (0.58)	2.84 (0.56)	2.74 (0.58)	2.54 (0.57)	0.20* (0.15)	7.3	0.97*	0.94* (0.82–0.98)	0.91* (0.25–0.98)
Ct.Po	3.46 (2.15)	3.35 (1.79)	3.87 (2.02)	3.56 (1.96)	2.94 (1.48)					
Log Ct.Po	1.07 (0.60)	1.07 (0.55)	1.22 (0.53)	1.12 (0.55)	0.95 (0.52)	0.18* (0.13)	16.1	0.97*	0.95* (0.84–0.98)	0.92* (0.25–0.98)
Ct.Po.Dm	0.18 (0.03)	0.17 (0.02)	0.18 (0.03)	0.18 (0.03)	0.17 (0.02)					
Log Ct.Po.Dm	− 1.74 (0.16)	− 1.76 (0.13)	− 1.71 (0.14)	− 1.74 (0.14)	− 1.80 (0.12)	0.06* (0.06)	3.5	0.89*	0.87* (0.78–0.92)	0.79* (0.26–0.92)
Ct.Ar	60.9 (15.4)	61.0 (15.6)	62.73 (15.82)	61.5 (15.6)	60.1 (15.6)	1.42* (1.20)	2.3	1.00*	0.99* (0.97–1.00)	0.99* (0.90–1.00)

Values are presented as mean (SD), unless stated otherwise. *p value < 0.001

*AUTO* automatic contouring method, *Ct.TV* cortical total volume in mm^3^, *Ct.BV* cortical bone volume in mm^3^, *Ct.Th* cortical thickness in mm, *Ct.vBMD* cortical vBMD in mgHA/cm^3^, *Ct. Po.V* cortical pore volume in mm^3^, *Ct.Po* cortical porosity in %, *Ct.Po.Dm* cortical pore diameter in mm, *Ct.Ar* cortical area in mm^2^, *ICC* intraclass correlation coefficient, *diff* difference, *OP1* operator 1 semi-automatic contouring method, *OP2* operator 2 semi-automatic contouring method, *OP3* operator 3 semi-automatic contouring method, *S-AUTOmean* mean value of the three independent operators

**Table 2:** Differences in mean values, correlations, and ICCs of the cortical bone parameters of the distal radius for the three independent operators

	Diff between OP1 and OP2	Diff between OP1 and OP3	Diff between OP2 and OP3	Diff between OP1 and OP2 (%)	Diff between OP1 and OP3 (%)	Diff between OP2 and OP3 (%)	Pearson’s *r* OP1–OP2	Pearson’s *r* OP1–OP3	Pearson’s *r* OP2–OP3	ICC (95%CI) OP1–OP2	ICC (95% CI) OP1–OP3	ICC (95% CI) OP2–OP3
Ct.TV	− 0.74 (9.28)	− 16.4** (13.6)	− 15.7** (13.7)	− 0.1	− 3.0	− 2.9	1.00**	1.00**	1.00**	1.00** (1.00–1.00)	0.99** (0.84–1.00)	0.99** (0.87–1.00)
Ct.BV	− 0.36 (4.59)	− 6.76** (6.06)	− 6.39** (6.00)	− 0.1	− 1.3	− 1.3	1.00**	1.00**	1.00**	1.00** (1.00–1.00)	1.00** (0.97–1.00)	1.00** (0.98–1.00)
Ct.Th	0.00 (0.01)	0.00 (0.01)	0.00** (0.01)	0.4	0.2	0.1	1.00**	1.00**	1.00**	1.00** (1.00–1.00)	1.00** (1.00–1.00)	1.00** (1.00–1.00)
Ct.vBMD	− 1.91 (9.69)	6.23** (8.36)	8.14** (8.38)	− 0.2	0.7	0.9	0.99**	0.99**	0.99**	0.99** (0.98–0.99)	0.99** (0.94–1.00)	0.98** (0.86–0.99)
Log Ct.Po.V	0.00 (0.14)	− 0.17** (0.15)	− 0.18** (0.15)	0.2	− 6.2	− 6.7	0.98**	0.97**	0.97**	0.96*** (0.93–0.98)	0.93*** (0.75–0.97)	0.92** (0.45–0.98)
Log Ct.Po	0.00 (0.12)	− 0.15** (0.14)	− 0.15** (0.14)	0.3	− 14.0	− 14.2	0.98**	0.98**	0.97**	0.94** (0.90–0.97)	0.94** (0.82–0.98)	0.93** (0.50–0.98)
Log Ct.Po.Dm	0.02* (0.06)	− 0.03* (0.08)	− 0.05** (0.07)	1.0	− 1.7	− 2.8	0.95**	0.88**	0.87**	0.91** (0.84–0.95)	0.85** (0.75–0.92)	0.82** (0.55–0.92)
Ct.Ar	− 0.08 (1.03)	− 1.82** (1.50)	− 1.74** (1.52)	− 0.1	− 3.0	− 2.9	1.00**	1.00**	1.00**	1.00** (1.00–1.00)	0.99** (0.84–1.00)	0.99** (0.87–1.00)

Values are presented as mean (SD), unless stated otherwise. *p value < 0.01, **p value < 0.001

*Ct.TV* cortical total volume in mm^3^, *Ct.BV* cortical bone volume in mm^3^, *Ct.Th* cortical thickness in mm, *Ct.vBMD* cortical vBMD in mgHA/cm^3^, *Ct. Po.V* cortical pore volume in mm^3^, *Ct.Po* cortical porosity in %, *Ct.Po.Dm* cortical pore diameter in mm, *Ct.Ar* cortical area in mm^2^, *OP1* operator 1 semi-automatic contouring method, *OP2* operator 2 semi-automatic contouring method, *OP3* operator 3 semi-automatic contouring method

**Table 3:** Mean values, differences in mean values, correlations, and ICC’s of the cortical bone parameters of the distal tibia

	OP1	OP2	OP3	S-AUTOmean	AUTO	Diff between S-AUTOmean and AUTO	Diff between S-AUTOmean and AUTO (%)	Pearson’s *r* S-AUTOmean–AUTO	ICC OP1–OP2–OP3 (95% CI)	ICC S-AUTOmean–AUTO (95% CI)
Ct.TV	1095.9 (272.9)	1121.1 (275.6)	1140.8 (287.3)	1119.3 (278.1)	1070.9 (273.5)	48.4** (40.9)	4.3	0.99**	0.99** (0.95–1.00)	0.97** (0.70–0.99)
Ct.BV	966.2 (245.1)	979.0 (245.3)	985.2 (250.8)	976.8 (246.9)	952.9 (246.8)	23.9** (21.6)	2.5	1.00**	1.00** (0.99–1.00)	0.99** (0.90–1.00)
Ct.Th	1.13 (0.25)	1.13 (0.25)	1.10 (0.23)	1.12 (0.24)	1.13 (0.26)	− 0.01 (0.04)	− 0.9	0.99**	0.99** (0.97–1.00)	0.99** (0.98–0.99)
Ct.vBMD	860.7 (63.7)	856.3 (65.9)	852.9 (65.1)	856.6 (64.8)	871.1 (56.6)	− 14.5** (13.8)	− 1.7	0.98**	0.99** (0.97–1.00)	0.95** (0.62–0.98)
Ct.Po.V	89.8 (37.0)	95.7 (40.1)	99.3 (40.3)	94.9 (38.8)	79.5 (32.6)					
Log Ct.Po.V	4.40 (0.48)	4.46 (0.48)	4.50 (0.46)	4.46 (0.47)	4.28 (0.45)	0.17** (0.14)	3.8	0.95**	0.97** (0.91–0.99)	0.89** (0.40–0.98)
Ct.Po	8.52 (3.00)	8.91 (3.19)	9.18 (3.08)	8.87 (3.07)	7.71 (2.50)					
Log Ct.Po	2.07 (0.39)	2.12 (0.39)	2.15 (0.37)	2.12 (0.38)	1.98 (0.36)	0.13** (0.11)	6.1	0.96**	0.98** (0.92–0.99)	0.90** (0.28–0.97)
Ct.Po.Dm	0.19 (0.02)	0.19 (0.02)	0.19 (0.02)	0.19 (0.02)	0.18 (0.02)					
Log Ct.Po.Dm	− 1.66 (0.09)	− 1.66 (0.09)	− 1.66 (0.09)	− 1.66 (0.09)	− 1.70 (0.08)	0.04** (0.05)	2.4	0.80**	0.95** (0.92–0.97)	0.74** (0.40–0.87)
Ct.Ar	121.5 (30.3)	124.3 (30.6)	126.5 (31.9)	124.1 (30.8)	118.7 (30.3)	5.36** (4.54)	4.3	0.99**	0.99** (0.95–1.00)	0.97** (0.70–0.99)

Values are presented as mean (SD), unless stated otherwise. *p value < 0.01, **p value < 0.001

*AUTO* automatic contouring method, *Ct.TV* cortical total volume in mm^3^, *Ct.BV* cortical bone volume in mm^3^, *Ct.Th* cortical thickness in mm, *Ct.vBMD* cortical vBMD in mgHA/cm^3^, *Ct. Po.V* cortical pore volume in mm^3^, *Ct.Po* cortical porosity in %, *Ct.Po.Dm* cortical pore diameter in mm, *Ct.Ar* cortical area in mm^2^, *ICC* intraclass correlation coefficient, *diff* difference, *OP1* operator 1 semi-automatic contouring method, *OP2* operator 2 semi-automatic contouring method, *OP3* operator 3 semi-automatic contouring method, *S-AUTOmean* mean value of the three independent operators

**Table 4:** Differences in mean values, correlations, and ICCs of the cortical bone parameters of the distal tibia for the three independent operators

	Diff between OP1 and OP2	Diff between OP1 and OP3	Diff between OP2 and OP3	Diff between OP1 and OP2, %	Diff between OP1 and OP3, %	Diff between OP2 and OP3, %	Pearson’s *r* OP1–OP2	Pearson’s *r* OP1–OP3	Pearson’s *r* OP2–OP3	ICC (95%CI) OP1–OP2	ICC (95% CI) OP1–OP3	ICC (95% CI) OP2–OP3
Ct.TV	− 25.2** (29.8)	− 44.8** (31.6)	− 19.7** (34.1)	− 2.3	− 4.1	− 1.8	0.99**	1.00**	0.99**	0.99** (0.95–1.00)	0.98** (0.62–1.00)	0.99** (0.97–1.00)
Ct.BV	− 12.8** (15.2)	− 19.0** (15.0)	− 6.19* (17.4)	− 1.3	− 2.0	− 0.6	1.00**	1.00**	1.00**	1.00** (0.98–1.00)	1.00** (0.92–1.00)	1.00** (1.00–1.00)
Ct.Th	− 0.00 (0.01)	0.02** (0.03)	0.03** (0.03)	− 0.0	2.4	2.7	1.00**	1.00**	1.00**	1.00** (1.00–1.00)	0.99** (0.90–1.00)	0.99** (0.89–1.00)
Ct.vBMD	4.35** (7.33)	7.76** (7.70)	3.40* (7.82)	0.5	0.9	0.4	0.99**	0.99**	0.99**	0.99** (0.98–1.00)	0.99** (0.88–1.00)	0.99** (0.98–1.00)
Log Ct.Po.V	− 0.06** (0.08)	− 0.11** (0.08)	− 0.04* (0.09)	− 1.4	− 2.5	− 0.9	0.99**	0.99**	0.98**	0.96** (0.88–0.98)	0.95** (0.60–0.98)	0.98** (0.95–0.99)
Log Ct.Po	− 0.04** (0.06)	− 0.08** (0.07)	− 0.04* (0.07)	− 1.9	− 3.9	− 1.9	0.99**	0.99**	0.98**	0.98** (0.92–0.99)	0.96** (0.63–0.99)	0.98** (0.95–0.99)
Log Ct.Po.Dm	0.00 (0.02)	0.00 (0.03)	0.00 (0.03)	0.1	0.2	0.1	0.97**	0.95**	0.93**	0.97** (0.94–0.98)	0.95** (0.91–0.97)	0.94** (0.89–0.96)
Ct.Ar	− 2.79** (3.24)	− 4.97** (3.51)	− 2.18** (3.78)	− 2.3	− 4.1	− 1.8	0.99**	1.00**	1.00**	0.99** (0.95–1.00)	0.98** (0.62–1.00)	0.99** (0.97–1.00)

Values are presented as mean (SD), unless stated otherwise. *p value < 0.01, **p value < 0.001

*Ct.TV* cortical total volume in mm^3^, *Ct.BV* cortical bone volume in mm^3^, *Ct.Th* cortical thickness in mm, *Ct.vBMD* cortical vBMD in mgHA/cm^3^, *Ct. Po.V* cortical pore volume in mm^3^, *Ct.Po* cortical porosity in %, *Ct.Po.Dm* cortical pore diameter in mm, *Ct.Ar* cortical area in mm^2^, *OP1* operator 1 semi-automatic contouring method, *OP2* operator 2 semi-automatic contouring method, *OP3* operator 3 semi-automatic contouring method

The percentage difference in cortical bone parameters of the distal radius between S-AUTOmean–AUTO, and between the pairs of operators was generally low (5% or less), except for Ct.Po.V and Ct.Po of the distal radius (Ct.Po.V: S-AUTOmean–AUTO 7.3%, OP1–OP3 − 6.2%, OP2–OP3 − 6.7%; Ct.Po: S-AUTOmean–AUTO 16.1%, OP1–OP3 − 14.0%, OP2–OP3 − 14.2%) and Ct.Po of the distal tibia (S-AUTOmean–AUTO 6.1%). The mean cortical bone parameters obtained by AUTO were generally slightly lower than the parameters obtained by the independent operators.

### Correlation Coefficients

Pearson’s *r* and the ICC of all bone parameters of the three operators (OP1–OP2–OP3) and of the two methods (S-AUTOmean–AUTO) are shown in Table 1 (radius) and Table 3 (tibia). Pearson’s *r* and the ICC of all bone parameters of the pairs of operators (OP1–OP2, OP1–OP3, and OP2–OP3) are shown in Table 2 (radius) and Table 4 (tibia).

The correlation coefficients for S-AUTOmean–AUTO and the pairs of operators were > 0.9 for almost all cortical bone parameters of both the distal radius and tibia (except for log Ct.Po.Dm of the radius: S-AUTOmean–AUTO 0.89, OP1–OP3 0.88, OP2–OP3 0.87, and log Ct.Po.Dm of the tibia: S-AUTOmean–AUTO 0.80). The ICC of S-AUTOmean and AUTO was high (> 0.81) for almost all cortical bone parameters of the distal radius and tibia (except for log Ct.Po.Dm of the radius 0.79 and log Ct.Po.Dm of the tibia 0.74). The ICC of OP1–OP2–OP3 and the pairs of operators was > 0.81 for all parameters of the distal radius and tibia.

### Precision Error

The RMS-CV% for all cortical bone parameters of both the distal radius and distal tibia are shown in Table 5 (OP1–OP2–OP3 and S-AUTOmean–AUTO) and Table 6 (pairs of operators). The precision error of the parameters Ct.TV, Ct.BV, Ct.Th, Ct.vBMD, and Ct.Ar was lower than the precision error of the porosity-related parameters (Ct.Po.V, Ct.Po, Ct.Po.Dm) for both the distal radius and tibia. The 95% confidence intervals of the precision errors of S-AUTOmean–AUTO of all cortical bone parameters of the distal radius had an overlap with the 95% confidence intervals of the precision errors of OP1–OP2–OP3, whereas the precision errors of S-AUTOmean–AUTO of the parameters of the distal radius were higher than those of OP1–OP2–OP3 (except for Ct.Th: OP1–OP2–OP3 1.26 (1.11–1.46), S-AUTOmean–AUTO 1.15 (0.96–1.43)).

**Table 5:** Root mean square coefficient of variation (RMS-CV%) of the cortical bone parameters of the distal radius and tibia

	Mean (SD) OP1/OP2/OP3	Mean (SD) S-AUTOmean/AUTO	RMS-CV% (95% CI) OP1/OP2/OP3	RMS-CV% (95% CI) S-AUTOmean/AUTO
Radius				
Ct.TV	555.1 (140.5)	548.7 (140.3)	2.39 (2.10–2.77)	2.50 (2.09–3.11)
Ct.BV	507.6 (137.0)	504.6 (137.0)	1.21 (1.06–1.41)	1.39 (1.16–1.73)
Ct.Th	0.84 (0.18)	0.84 (0.18)	1.26 (1.11–1.46)	1.15 (0.96–1.43)
Ct.vBMD	943.8 (58.7)	948.5 (55.9)	0.85 (0.74–0.99)	1.15 (0.97–1.44)
Ct.Po.V	18.2 (10.7)	16.5 (9.75)	14.3 (12.5–16.6)	17.2 (14.4–21.4)
Ct.Po	3.56 (1.96)	3.25 (1.69)	12.9 (11.3–15.0)	15.3 (12.8–19.0)
Ct.Po.Dm	0.18 (0.03)	0.17 (0.02)	5.25 (4.61–6.10)	6.21 (5.20–7.72)
Ct.Ar	61.5 (15.6)	60.8 (15.6)	2.39 (2.10–2.77)	2.50 (2.09–3.11)
Tibia				
Ct.TV	1119.3 (278.1)	1095.1 (275.1)	2.68 (2.36–3.11)	4.09 (3.42–5.08)
Ct.BV	976.8 (246.9)	964.9 (246.6)	1.47 (1.29–1.71)	2.44 (2.04–3.03)
Ct.Th	1.12 (0.24)	1.13 (0.25)	1.94 (1.71–2.25)	1.81 (1.52–2.25)
Ct.vBMD	856.6 (64.8)	863.9 (60.4)	0.80 (0.71–0.93)	1.73 (1.44–2.14)
Ct.Po.V	94.9 (38.8)	87.2 (35.0)	7.99 (7.02–9.28)	15.5 (13.0-19.3)
Ct.Po	8.87 (3.07)	8.29 (2.75)	6.19 (5.44–7.19)	12.1 (10.1–15.0)
Ct.Po.Dm	0.19 (0.02)	0.19 (0.02)	1.97 (1.73–2.28)	4.56 (3.82–5.67)
Ct.Ar	124.1 (30.8)	121.4 (30.5)	2.68 (2.36–3.11)	4.09 (3.42–5.08)

*Ct.TV* cortical total volume in mm^3^, *Ct.BV* cortical bone volume in mm^3^, *Ct.Th* cortical thickness in mm, *Ct.vBMD* cortical vBMD in mgHA/cm^3^, *Ct. Po.V* cortical pore volume in mm^3^, *Ct.Po* cortical porosity in %; *Ct.Po.Dm* cortical pore diameter in mm; *Ct.Ar* cortical area in mm^2^, *OP1* operator 1 semi-automatic contouring method, *OP2* operator 2 semi-automatic contouring method, *OP3* operator 3 semi-automatic contouring method, *S-AUTOmean* mean value of the three independent operators, *AUTO* automatic contouring method

**Table 6:** Root mean square coefficient of variation of the cortical bone parameters of the distal radius and tibia for the independent operators

	Mean (SD) OP1–OP2	Mean (SD) OP1–OP3	Mean (SD) OP2–OP3	RMS-CV% (95% CI) OP1–OP2	RMS-CV% (95% CI) OP1–OP3	RMS-CV% (95% CI) OP2–OP3
Radius						
Ct.TV	549.8 (139.6)	557.6 (140.5)	558.0 (141.6)	1.48 (1.24–1.84)	2.66 (2.22–3.30)	2.77 (2.32–3.44)
Ct.BV	505.4 (136.7)	508.6 (136.8)	508.8 (137.5)	0.91 (0.76–1.13)	1.29 (1.08–1.61)	1.38 (1.15–1.71)
Ct.Th	0.84 (0.18)	0.84 (0.18)	0.84 (0.18)	1.51 (1.26–1.88)	1.10 (0.92–1.37)	1.09 (0.91–1.36)
Ct.vBMD	946.2 (58.7)	942.1 (59.8)	943.1 (57.9)	0.81 (0.68–1.01)	0.82 (0.69–1.02)	0.92 (0.77–1.14)
Ct.Po.v	17.3 (10.4)	18.7 (11.0)	18.5 (10.7)	9.48 (7.93–11.8)	15.51 (13.0–19.3)	15.9 (13.3–19.8)
Ct.Po	3.40 (1.95)	3.67 (2.07)	3.61 (1.89)	8.41 (7.04–10.5)	14.2 (11.9–17.6)	14.4 (12.1–17.9)
Ct.Po.Dm	0.18 (0.03)	0.18 (0.03)	0.18 (0.02)	4.03 (3.37–5.01)	5.65 (4.73–7.02)	5.82 (4.87–7.24)
Ct.Ar	61.0 (15.5)	61.8 (15.6)	61.9 (15.7)	1.48 (1.24–1.84)	2.66 (2.22–3.30)	2.77 (2.32–3.44)
Tibia						
Ct.TV	1108.5 (273.9)	1118.4 (279.8)	1130.9 (281.0)	2.42 (2.03–3.01)	3.31 (2.77–4.11)	2.18 (1.83–2.71)
Ct.BV	972.6 (245.1)	975.7 (247.8)	982.1 (247.9)	1.49 (1.25–1.86)	1.70 (1.43–2.12)	1.18 (0.99–1.46)
Ct.Th	1.13 (0.25)	1.12 (0.24)	1.12 (0.24)	0.75 (0.62–0.93)	2.30 (1.92–2.86)	2.37 (1.98–2.94)
Ct.vBMD	858.2 (64.7)	856.8 (64.3)	854.6 (65.4)	0.74 (0.62–0.92)	0.94 (0.79–1.17)	0.71 (0.60–0.89)
Ct.Po.v	92.8 (38.3)	94.5 (38.4)	97.5 (39.9)	7.14 (5.98–8.88)	9.48 (7.93–11.8)	7.09 (5.93–8.81)
Ct.Po	8.72 (3.08)	8.85 (3.03)	9.05 (3.12)	5.37 (4.50–6.68)	7.39 (6.18–9.19)	5.59 (4.67–6.94)
Ct.Po.Dm	0.19 (0.02)	0.19 (0.02)	0.19 (0.02)	1.68 (1.41–2.09)	1.97 (1.65–2.45)	2.18 (1.83–2.71)
Ct.Ar	122.9 (30.4)	124.0 (31.0)	125.4 (31.2)	2.42 (2.03–3.01)	3.31 (2.77–4.11)	2.18 (1.83–2.72)

*Ct.TV* cortical total volume in mm^3^, *Ct.BV* cortical bone volume in mm^3^, *Ct.Th* cortical thickness in mm, *Ct.vBMD* cortical vBMD in mgHA/cm^3^, *Ct. Po.V* cortical pore volume in mm^3^, *Ct.Po* cortical porosity in %, *Ct.Po.Dm* cortical pore diameter in mm, *Ct.Ar* cortical area in mm^2^, *OP1* operator 1 semi-automatic contouring method, *OP2* operator 2 semi-automatic contouring method, *OP3* operator 3 semi-automatic contouring method

### Bland–Altman Plots

**Fig. 1:**
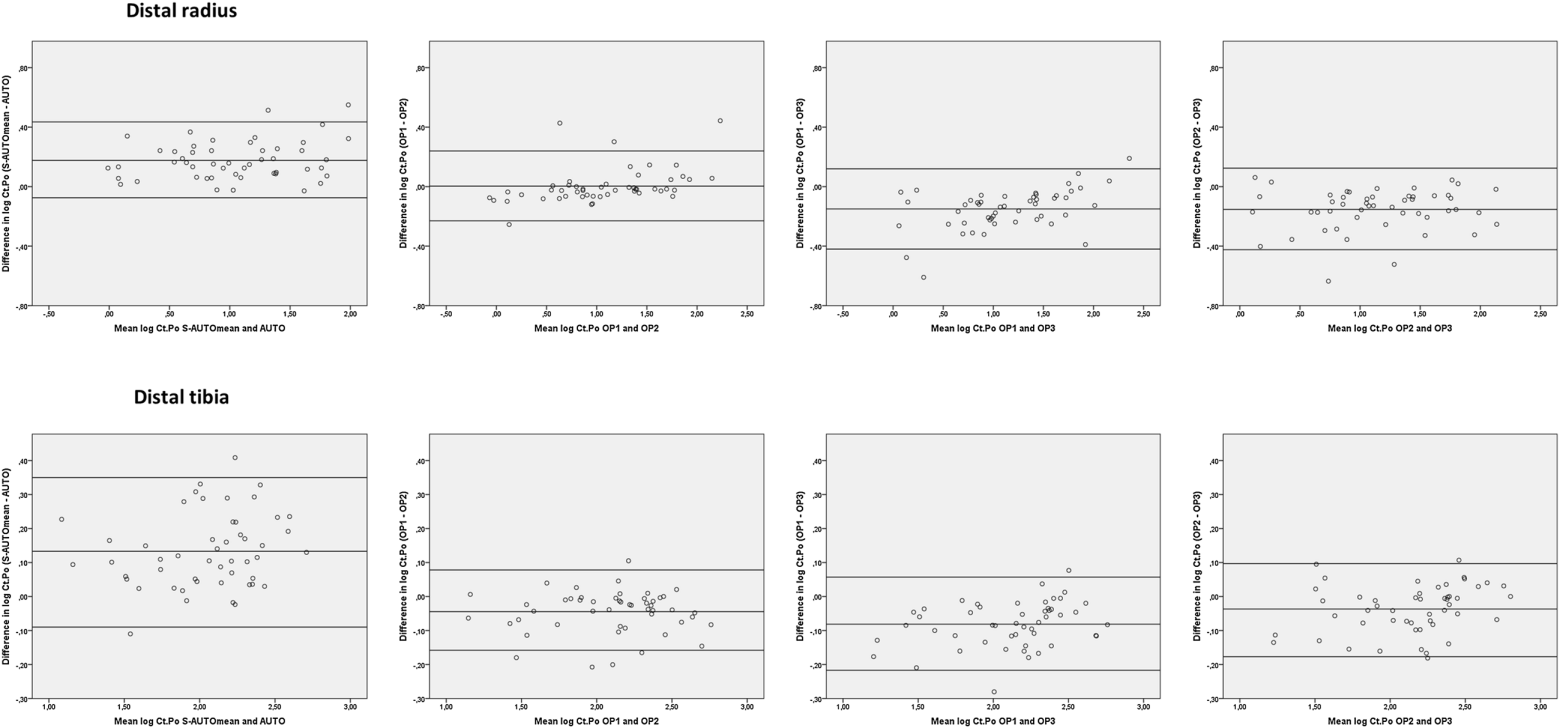
Bland–Altman plots for the parameter cortical porosity of both the distal radius (top row) and tibia (lower row). First column: S-AUTOmean–AUTO. Second column: operator 1–operator 2. Third column: operator 2–operator 3. Fourth column: operator 2–operator 3. The limits of agreement were calculated as mean ± 1.96* SD. *AUTO* automatic contouring method, *Ct.Po* cortical porosity in %, *OP1* operator 1 semi-automatic contouring method, *OP2* operator 2 semi-automatic contouring method, *OP3* operator 3 semi-automatic contouring method, *S-AUTOmean* mean value of the three independent operators

The Bland–Altman plots showed no systematic error in any of the plots of S-AUTOmean–AUTO (Fig. 1 (cortical porosity of the radius and tibia); Supplemental Figs. 1 (radius) and 2 (tibia)) and of the individual operators (Fig. 1 (cortical porosity of the radius and tibia); Supplemental Figs. 3 (radius) and 4 (tibia)). The 95% confidence intervals were the widest in the plots of S-AUTOmean–AUTO, displaying more variability in error between S-AUTOmean and AUTO than between OP1 and OP2, OP1 and OP3, and OP2 and OP3. Additionally, use of the AUTO method instead of the S-AUTO method resulted in lower absolute values of the porosity-related parameters, while also clear variability in determined cortical porosity between the individual operators was observed (Fig. 1).

**Fig. 2:**
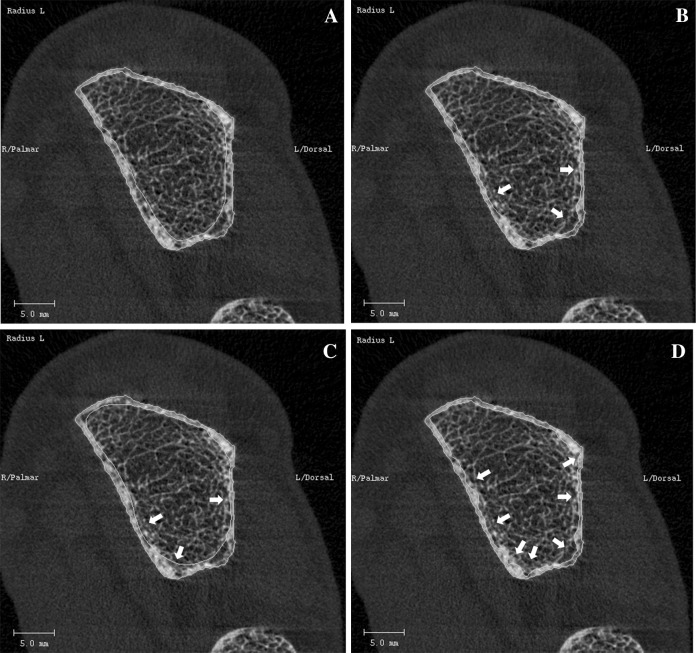
Slice 75 of an HR-pQCT scan of the distal radius, as modified by operator 1 (**a**), operator 2 (**b**), operator 3 (**c**), and the automatic method (**d**). The arrows indicate the differences in the endocortical contour of operator 2, operator 3, and the automatic method, compared to operator 1. The presented scan is the outlier in the Bland–Altman plots of the distal radius of S-AUTOmean–AUTO and OP1–OP2 for the parameters Ct.TV, Ct.BV, Ct.Th, Ct.vBMD, and Ct.Ar

One outlier (> 2 SD from mean) was observed in the Bland–Altman plots of the distal radius of S-AUTOmean–AUTO and OP1–OP2 for the parameters Ct.TV, Ct.BV, Ct.Th, Ct.vBMD, and Ct.Ar. Slice 75 of this specific scan is shown in Fig. 2. Clear differences in the location of the endocortical contours of AUTO, OP2, and OP3 are visible when compared to the contour of OP1.

## Discussion

In this study, we evaluated whether the AUTO contour instead of the S-AUTO contour could be used for cortical bone analysis in a cross-sectional study. Therefore, the (average) results obtained with the AUTO method were compared to the average results of the S-AUTO method. Additionally, variability in results as obtained with the different S-AUTO contours was examined.

The smallest differences in mean cortical bone parameters, the highest ICCs and lowest precision error were found between independent operators. Additionally, the precision error of the three operators was generally lower than the precision error of S-AUTOmean and AUTO. This indicates that correction of the contours, even when the scans are analyzed by several independent operators, introduces a smaller error than the use of the uncorrected, automatically generated contours. However, the mean differences in cortical bone parameters between S-AUTOmean and AUTO were highly comparable to the differences between OP1 and OP3. The absolute level of agreement between S-AUTOmean and AUTO was high and the Bland–Altman plots of the differences between S-AUTOmean and AUTO in cortical parameters showed no systematic error.

The precision errors of the cortical bone parameters of the distal radius of OP1–OP3 and of OP2–OP3 were generally comparable to those of S-AUTOmean–AUTO. In contrast, the precision errors of the cortical bone parameters of the distal tibia of S-AUTOmean and AUTO were higher than the precision error of the individual operators, particularly for the porosity-related parameters. Compared to precision errors for cortical bone parameters obtained by a short-term repositioning study that used S-AUTO [10], the precision errors of S-AUTOmean and AUTO were higher, particularly those of Ct.Po.V and Ct.Po of the distal tibia. In contrast, the error introduced by not correcting the endocortical contour to the precision was comparable to the error introduced by variability in positioning of the reference line for the parameters Ct.BMD and Ct.Th, but not for Ct.Po [22]. Additionally, the precision error of S-AUTOmean and AUTO for Ct.Po and Ct.Po.Dm of the distal radius is comparable to the precision error of a donor specimen that was scanned and evaluated at 9 different sites [23]. Although it is known that motion of the subject has a large influence on the precision error of densitometry and trabecular microarchitectural parameters [14], the influence of motion of the subject on cortical bone parameters has not been examined. Thus, the AUTO method seems to introduce about the same error to the precision error as variability in the position of the reference line and as multicenter scanning.

Although all operators underwent the same training, clear differences between the endocortical contours of the three operators were visible (Fig. 2). The explanation for this difference is probably the gradual transition from cortical to trabecular bone, which makes identification of the endocortical border challenging [1, 9]. As a result of the presence of this transitional zone, each operator ‘sees’ his or her own truth and modifies the AUTO contour in a slightly different way, thereby including variable parts of the transitional zone. Currently, there are no studies published that compare different endocortical contours with histology (the golden standard), and it is thus currently unknown which contour marks the endocortical border correctly. Since the porosity of the trabecular compartment is much higher than the porosity of the cortical compartment, variability in inclusion of the transitional zone will influence the observed cortical bone parameters and will lead to an increased RMS-CV%, particularly of the porosity-related parameters. As shown in Fig. 2, the endocortical contour created by AUTO was closer to the periosteal border than the contour created by the independent operators, and thus included a smaller part of the transitional zone. As a result, use of AUTO results in lower absolute values of cortical porosity when compared to using the S-AUTO method (Fig. 1). This is in agreement with the study by Kawalilak et al., who also showed that the S-AUTO method resulted in a larger trabecularized cortex compared to the AUTO method [12]. Studies using AUTO contours instead of correct contours should therefore take into account that this will lead to lower absolute values of all parameters, except for the cortical vBMD which will be higher.

Limitations of our study include the generalizability; the mean age of our study population was 58 years, and therefore most of the included women will be postmenopausal. It may be expected that the AUTO method will also be reliable in premenopausal women because the endocortical border is often better recognizable in younger women [1]. Additionally, a study by Kawalilak et al. showed that the reproducibility of cortical bone parameters when the AUTO contour is used is better in premenopausal than in postmenopausal women [12]. In contrast, recognition of the endocortical border is more difficult in older individuals, which may lead to problems with both the AUTO and S-AUTP method. Future studies are warranted to examine the validity of the use of the AUTO method in both younger and older study populations. A second limitation of our study is the lack of scan–rescan data. We were therefore not able to determine the short- and/or long-term reproducibility of the uncorrected and corrected contours. However, a previous study showed a high reproducibility of both the AUTO method and the corrected contours [12]. Third, the study was not designed for comparing the two methods for a clinical outcome such as fracture prediction or treatment of osteoporosis. Future studies are warranted to examine whether the AUTO contour can also be used in studies with clinical outcomes. Fourth, for the examination of the cortical compartment, we used the method provided by the manufacturer. Therefore, all the results in this study are therefore only valid for this method, and cannot be extrapolated to other algorithms such as the commercially available StrAx© software [24]. Finally, the reference line for the HR-pQCT scans was placed at a fixed reference point, which resulted in the scanning of the same region in every participant. However, bone morphology at that region differs between individual patients, where a higher amount of cortical bone will be present in participants with relatively short extremities. Therefore, recent studies suggest scanning at a percentage distance of the total length of the bone [25, 26].

In conclusion, the S-AUTO method resulted in better reliability of the cortical bone parameters than the AUTO method in a cross-sectional study. However, it was shown that correction of the AUTO contour by different operators resulted in clear variability in cortical parameters. Additionally, the percent differences in cortical parameters, the ICCs, and the precision errors of the radius between the uncorrected automatically generated contour and the corrected contour were highly comparable to the errors observed between independent operators and no systematic error was observed in the Bland–Altman plots. Therefore, we believe that the AUTO contour can be used for cortical bone analysis in a cross-sectional study, although it should be taken into account that use of the AUTO instead of the S-AUTO method will result in lower absolute values of particularly the porosity-related parameters. The lower cortical parameters with AUTO and the variability in parameters between the individual operators can be explained by variable inclusion of the transitional zone.

## Electronic supplementary material

Below is the link to the electronic supplementary material.

**Supplemental Fig. 1** Bland–Altman plots for all cortical bone parameters of the distal radius for S-AUTOmean – AUTO. All parameters showed normal error distributions. The limits of agreement were calculated as mean ± 1.96* SD. AUTO, automatic contouring method; Ct.TV, cortical total volume in mm^3^; Ct.BV, cortical bone volume in mm^3^; Ct.Th, cortical thickness in mm; Ct.vBMD, cortical vBMD in mgHA/cm^3^; Ct. Po.V, cortical pore volume in mm^3^; Ct.Po.Dm, cortical pore diameter in mm; Ct.Ar, cortical area in mm^2^; S-AUTOmean, mean value of the three independent operators (TIF 100 KB)

**Supplemental Fig. 2** Bland–Altman plots for all cortical bone parameters of the distal tibia for S-AUTOmean – AUTO. All parameters showed normal error distributions. The limits of agreement were calculated as mean ± 1.96* SD. AUTO, automatic contouring method; Ct.TV, cortical total volume in mm^3^; Ct.BV, cortical bone volume in mm^3^; Ct.Th, cortical thickness in mm; Ct.vBMD, cortical vBMD in mgHA/cm^3^; Ct. Po.V, cortical pore volume in mm^3^; Ct.Po.Dm, cortical pore diameter in mm; Ct.Ar, cortical area in mm^2^; S-AUTOmean, mean value of the three independent operators (TIF 94 KB)

**Supplemental Fig. 3** Bland–Altman plots for all cortical bone parameters of the distal radius. Top row: operator 1 – operator 2. Middle row: operator 1 – operator 3. Lower row: operator 2 – operator 3. All parameters showed normal error distributions. The limits of agreement were calculated as mean ± 1.96* SD. Ct.TV, cortical total volume in mm^3^; Ct.BV, cortical bone volume in mm^3^; Ct.Th, cortical thickness in mm; Ct.vBMD, cortical vBMD in mgHA/cm^3^; Ct. Po.V, cortical pore volume in mm^3^; Ct.Po.Dm, cortical pore diameter in mm; Ct.Ar, cortical area in mm^2^; OP1, operator 1 semi-automatic contouring method; OP2, operator 2 semi-automatic contouring method; OP3, operator 3 semi-automatic contouring method (TIF 523 KB)

**Supplemental Fig. 3** Bland–Altman plots for all cortical bone parameters of the distal radius. Top row: operator 1 – operator 2. Middle row: operator 1 – operator 3. Lower row: operator 2 – operator 3. All parameters showed normal error distributions. The limits of agreement were calculated as mean ± 1.96* SD. Ct.TV, cortical total volume in mm^3^; Ct.BV, cortical bone volume in mm^3^; Ct.Th, cortical thickness in mm; Ct.vBMD, cortical vBMD in mgHA/cm^3^; Ct. Po.V, cortical pore volume in mm^3^; Ct.Po.Dm, cortical pore diameter in mm; Ct.Ar, cortical area in mm^2^; OP1, operator 1 semi-automatic contouring method; OP2, operator 2 semi-automatic contouring method; OP3, operator 3 semi-automatic contouring method (TIF 105 KB)

**Supplemental Fig. 4** Bland–Altman plots for all cortical bone parameters of the distal tibia. Top row: operator 1 – operator 2. Middle row: operator 1 – operator 3. Lower row: operator 2 – operator 3. All parameters showed normal error distributions. The limits of agreement were calculated as mean ± 1.96* SD. Ct.TV, cortical total volume in mm^3^; Ct.BV, cortical bone volume in mm^3^; Ct.Th, cortical thickness in mm; Ct.vBMD, cortical vBMD in mgHA/cm^3^; Ct. Po.V, cortical pore volume in mm^3^; Ct.Po.Dm, cortical pore diameter in mm; Ct.Ar, cortical area in mm^2^; OP1, operator 1 semi-automatic contouring method; OP2, operator 2 semi-automatic contouring method; OP3, operator 3 semi-automatic contouring method (TIF 541 KB)

**Supplemental Fig. 4** Bland–Altman plots for all cortical bone parameters of the distal tibia. Top row: operator 1 – operator 2. Middle row: operator 1 – operator 3. Lower row: operator 2 – operator 3. All parameters showed normal error distributions. The limits of agreement were calculated as mean ± 1.96* SD. Ct.TV, cortical total volume in mm^3^; Ct.BV, cortical bone volume in mm^3^; Ct.Th, cortical thickness in mm; Ct.vBMD, cortical vBMD in mgHA/cm^3^; Ct. Po.V, cortical pore volume in mm^3^; Ct.Po.Dm, cortical pore diameter in mm; Ct.Ar, cortical area in mm^2^; OP1, operator 1 semi-automatic contouring method; OP2, operator 2 semi-automatic contouring method; OP3, operator 3 semi-automatic contouring method (TIF 106 KB)
