# Supplementary figures and images for: Reliability of HR-pQCT Derived Cortical Bone Structural Parameters When Using Uncorrected Instead of Corrected Automatically Generated Endocortical Contours in a Cross-Sectional Study: The Maastricht Study
Source: Calcif Tissue Int. 2018 Mar 29;103(3):252–65. doi: 10.1007/s00223-018-0416-2 (PMC6105151; doi:10.1007/s00223-018-0416-2)

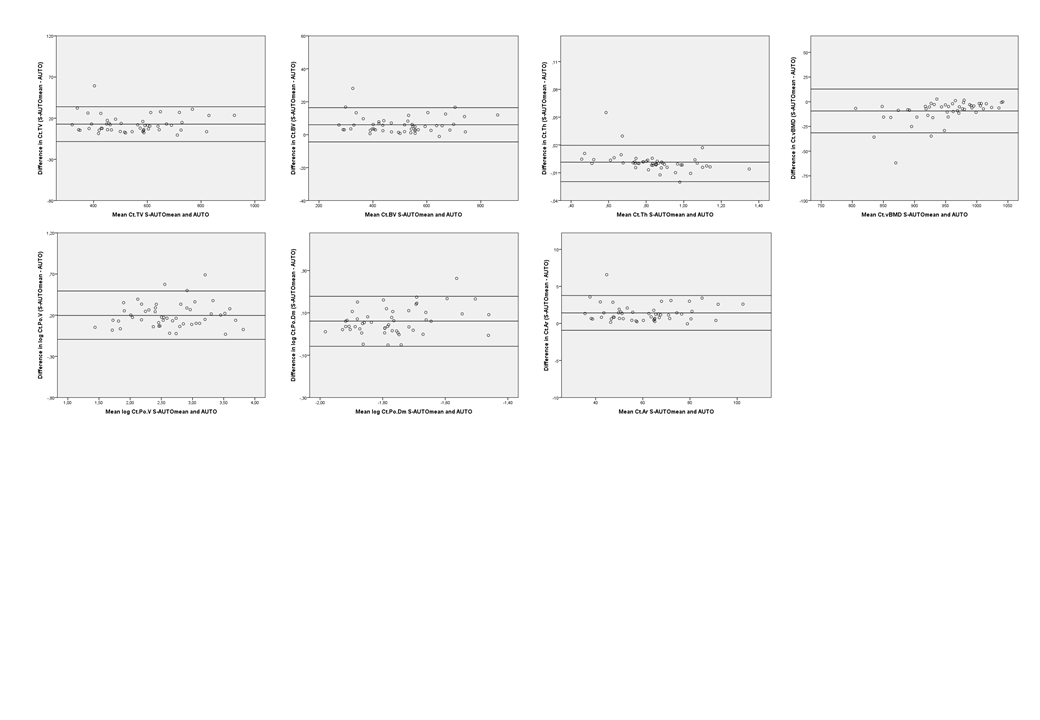

Supplement: Supplementary file 1 — Supplemental Fig. 1 Bland–Altman plots for all cortical bone parameters of the distal radius for S-AUTOmean – AUTO. All parameters showed normal error distributions. The limits of agreement were calculated as mean ± 1.96* SD. AUTO, automatic contouring method; Ct.TV, cortical total volume in mm3; Ct.BV, cortical bone volume in mm3; Ct.Th, cortical thickness in mm; Ct.vBMD, cortical vBMD in mgHA/cm3; Ct. Po.V, cortical pore volume in mm3; Ct.Po.Dm, cortical pore diameter in mm; Ct.Ar, cortical area in mm2; S-AUTOmean, mean value of the three independent operators (TIF 100 KB) [file 223_2018_416_MOESM1_ESM.tif]

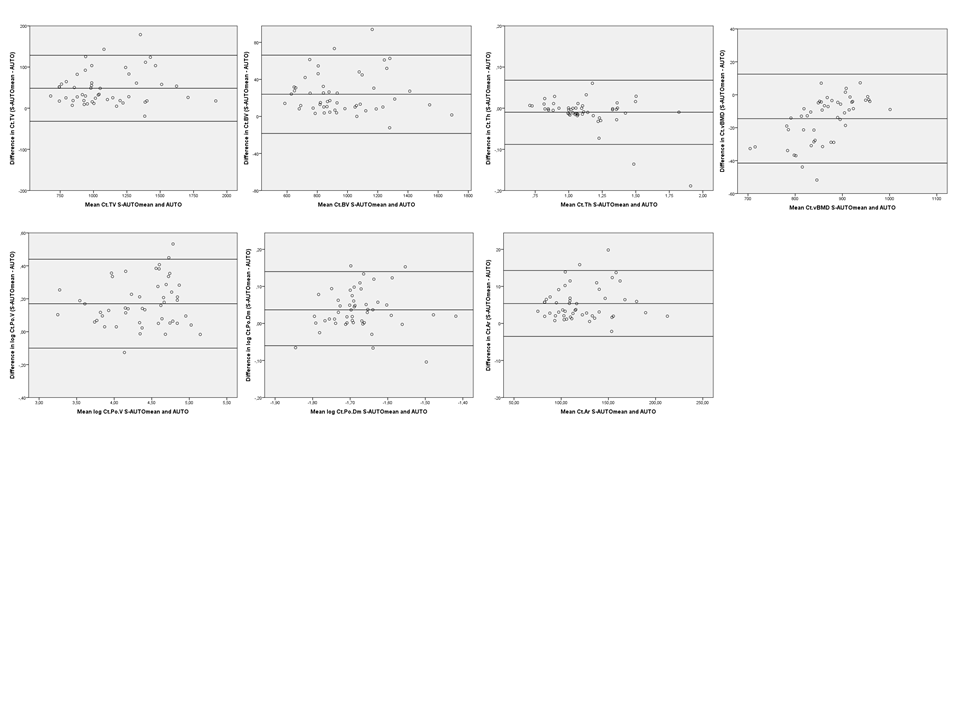

Supplement: Supplementary file 2 — Supplemental Fig. 2 Bland–Altman plots for all cortical bone parameters of the distal tibia for S-AUTOmean – AUTO. All parameters showed normal error distributions. The limits of agreement were calculated as mean ± 1.96* SD. AUTO, automatic contouring method; Ct.TV, cortical total volume in mm3; Ct.BV, cortical bone volume in mm3; Ct.Th, cortical thickness in mm; Ct.vBMD, cortical vBMD in mgHA/cm3; Ct. Po.V, cortical pore volume in mm3; Ct.Po.Dm, cortical pore diameter in mm; Ct.Ar, cortical area in mm2; S-AUTOmean, mean value of the three independent operators (TIF 94 KB) [file 223_2018_416_MOESM2_ESM.tif]

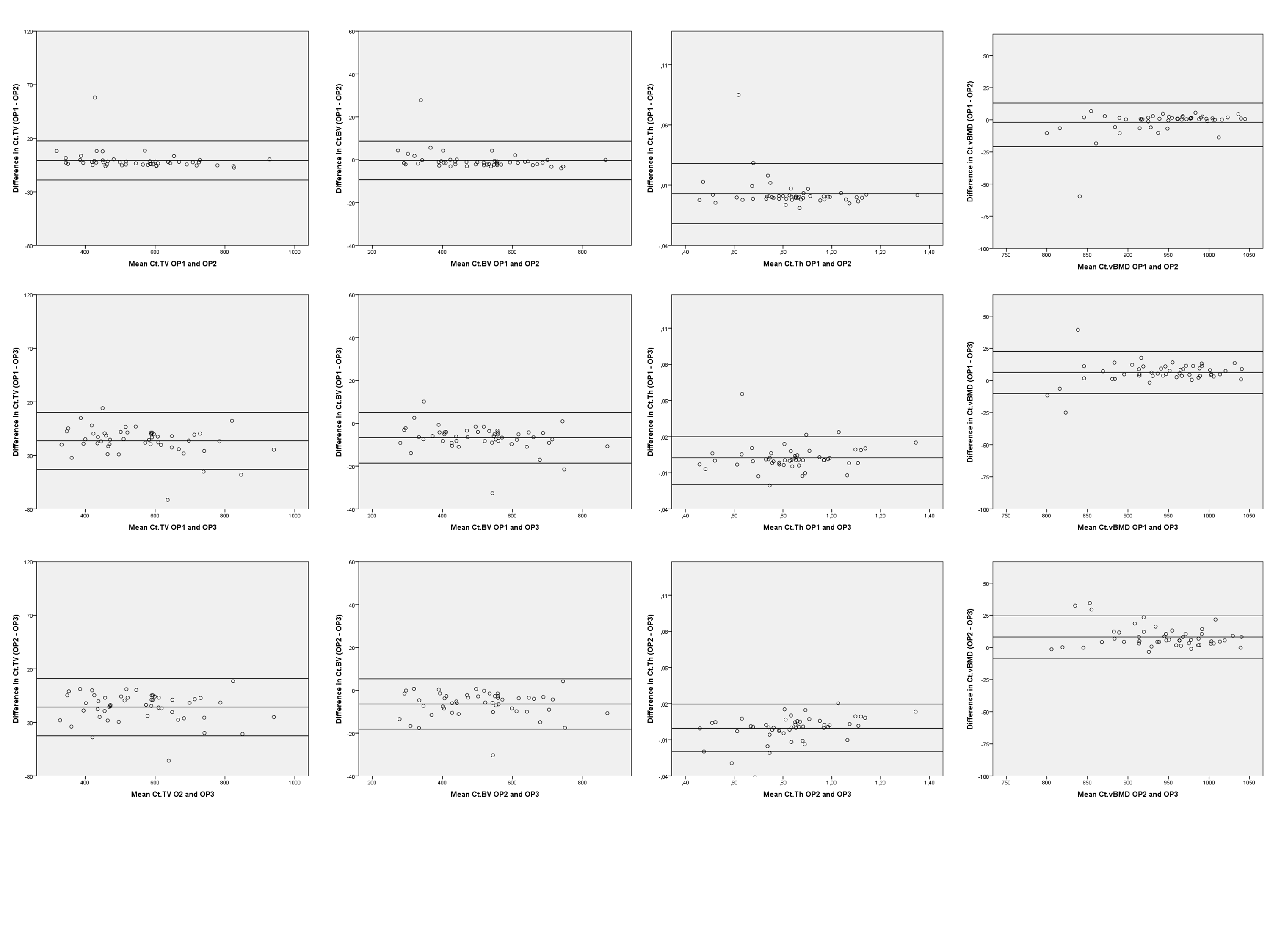

Supplement: Supplementary file 3 — Supplemental Fig. 3 Bland–Altman plots for all cortical bone parameters of the distal radius. Top row: operator 1 – operator 2. Middle row: operator 1 – operator 3. Lower row: operator 2 – operator 3. All parameters showed normal error distributions. The limits of agreement were calculated as mean ± 1.96* SD. Ct.TV, cortical total volume in mm3; Ct.BV, cortical bone volume in mm3; Ct.Th, cortical thickness in mm; Ct.vBMD, cortical vBMD in mgHA/cm3; Ct. Po.V, cortical pore volume in mm3; Ct.Po.Dm, cortical pore diameter in mm; Ct.Ar, cortical area in mm2; OP1, operator 1 semi-automatic contouring method; OP2, operator 2 semi-automatic contouring method; OP3, operator 3 semi-automatic contouring method (TIF 523 KB) [file 223_2018_416_MOESM3_ESM.tif]

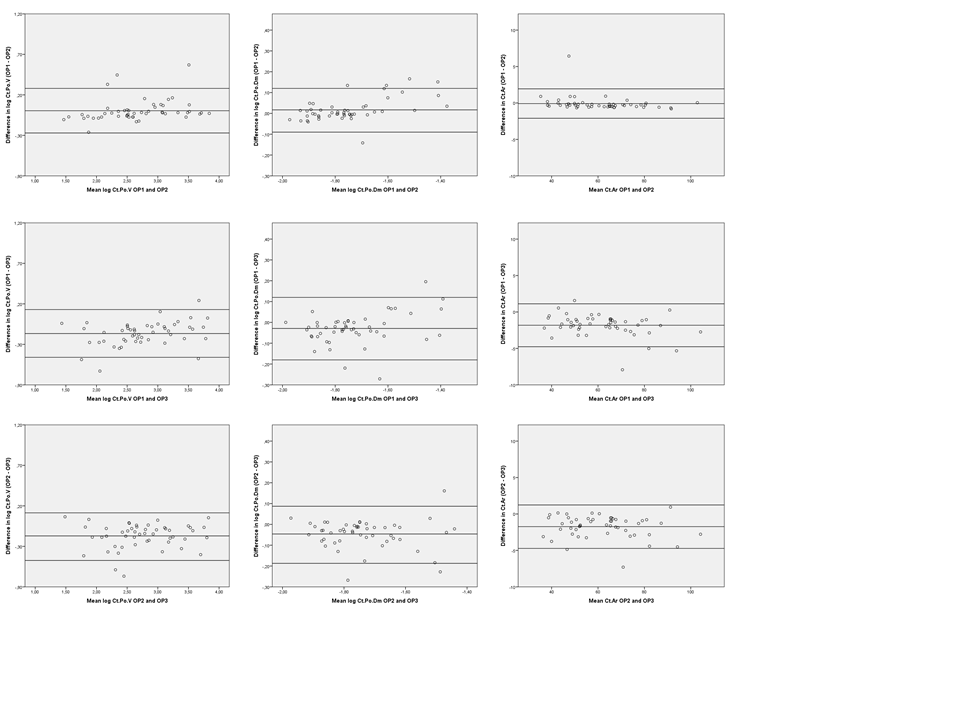

Supplement: Supplementary file 4 — Supplemental Fig. 3 Bland–Altman plots for all cortical bone parameters of the distal radius. Top row: operator 1 – operator 2. Middle row: operator 1 – operator 3. Lower row: operator 2 – operator 3. All parameters showed normal error distributions. The limits of agreement were calculated as mean ± 1.96* SD. Ct.TV, cortical total volume in mm3; Ct.BV, cortical bone volume in mm3; Ct.Th, cortical thickness in mm; Ct.vBMD, cortical vBMD in mgHA/cm3; Ct. Po.V, cortical pore volume in mm3; Ct.Po.Dm, cortical pore diameter in mm; Ct.Ar, cortical area in mm2; OP1, operator 1 semi-automatic contouring method; OP2, operator 2 semi-automatic contouring method; OP3, operator 3 semi-automatic contouring method (TIF 105 KB) [file 223_2018_416_MOESM4_ESM.tif]

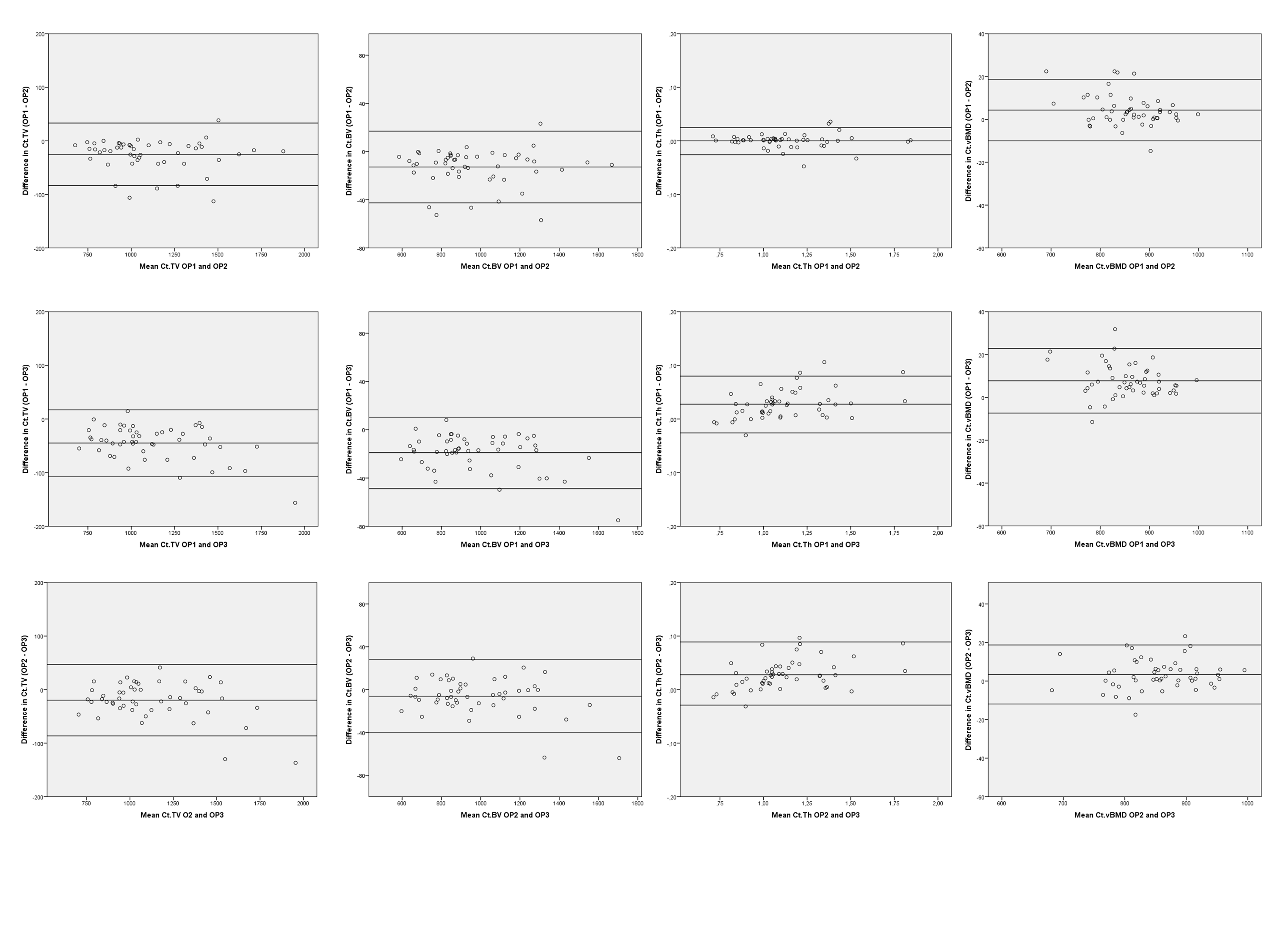

Supplement: Supplementary file 5 — Supplemental Fig. 4 Bland–Altman plots for all cortical bone parameters of the distal tibia. Top row: operator 1 – operator 2. Middle row: operator 1 – operator 3. Lower row: operator 2 – operator 3. All parameters showed normal error distributions. The limits of agreement were calculated as mean ± 1.96* SD. Ct.TV, cortical total volume in mm3; Ct.BV, cortical bone volume in mm3; Ct.Th, cortical thickness in mm; Ct.vBMD, cortical vBMD in mgHA/cm3; Ct. Po.V, cortical pore volume in mm3; Ct.Po.Dm, cortical pore diameter in mm; Ct.Ar, cortical area in mm2; OP1, operator 1 semi-automatic contouring method; OP2, operator 2 semi-automatic contouring method; OP3, operator 3 semi-automatic contouring method (TIF 541 KB) [file 223_2018_416_MOESM5_ESM.tif]

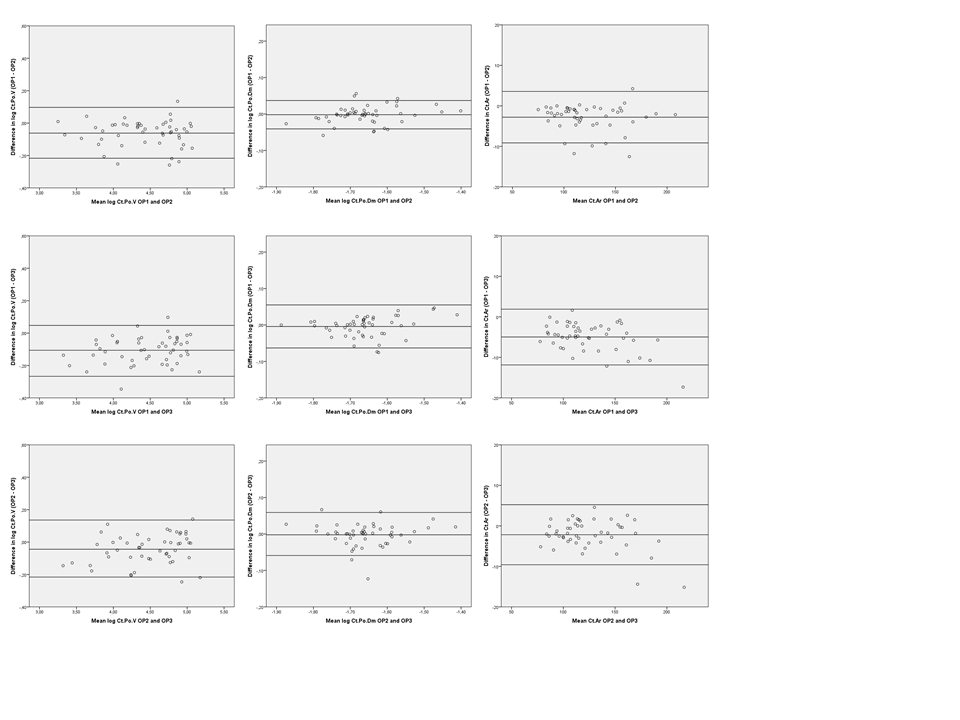

Supplement: Supplementary file 6 — Supplemental Fig. 4 Bland–Altman plots for all cortical bone parameters of the distal tibia. Top row: operator 1 – operator 2. Middle row: operator 1 – operator 3. Lower row: operator 2 – operator 3. All parameters showed normal error distributions. The limits of agreement were calculated as mean ± 1.96* SD. Ct.TV, cortical total volume in mm3; Ct.BV, cortical bone volume in mm3; Ct.Th, cortical thickness in mm; Ct.vBMD, cortical vBMD in mgHA/cm3; Ct. Po.V, cortical pore volume in mm3; Ct.Po.Dm, cortical pore diameter in mm; Ct.Ar, cortical area in mm2; OP1, operator 1 semi-automatic contouring method; OP2, operator 2 semi-automatic contouring method; OP3, operator 3 semi-automatic contouring method (TIF 106 KB) [file 223_2018_416_MOESM6_ESM.tif]
